# Eyes and Cold Water

**Published:** 1874-03

**Authors:** 


### EYES AND COLD WATER.

The aquatic furor has become so general, that for the simple reason that cold water is a pure, natural product, it is claimed to be a universal and beneficial application. Arsenic is a pure, natural and simple product; so is prussic acid, as obtained from a peach kernel. A single drop of tobacco oil will kill a cat or dog in five minutes.

Many persons are daily ruining their eyes by opening them in cold water of mornings. Cold water will harden and roughen the hands, and much more will it do so to the manyfold more delicate covering of the eye; *or*, the eye will, in self-defence, become scaly in the manner of a fish; that is, the coats of the eye will thicken, constituting a species of cateract, which must impair the sight. That water, cold and harsh as it is, should be applied to the eye for curative purposes, in place of that soft, warm, lubricating fluid which nature manufactures just for such purposes, indicates great thoughtlessness or great mental obliquity. Nothing stronger than luke warm water should ever be applied to the eye, except by special medical advice, and under special medical supervision; for we have only one pair to lose. Even warm water should be applied only by closing the eye and flapping it against the lid with the hand, patiently, scarcely letting the fingers touch the lid. This cools the eye more rapidly than cold water does, and without the shock, while its soothing effect is delightful, dissolving or washing out the yellow or other matter which may have accumulated over night, in half the time required by cold water.

